# Comparison of opioid local anesthetic combination regimens using the number of self-administrated boluses in patient-controlled epidural analgesia after cesarean section: A retrospective single-center study

**DOI:** 10.1097/MD.0000000000025560

**Published:** 2021-04-30

**Authors:** Go Otao, Toyoaki Maruta, Isao Tsuneyoshi

**Affiliations:** Department of Anesthesiology, University of Miyazaki Hospital, Miyazaki, Japan.

**Keywords:** cesarean section, opioids, patient-controlled epidural analgesia

## Abstract

The aim of this study was to assess the efficacy of combined opioids by comparing four regimens of patient-controlled epidural analgesia (PCEA) after cesarean section.

Parturient patients who underwent elective or emergent cesarean section under combined spinal and epidural anesthesia from April 2013 to March 2016 were retrospectively analyzed. Based on PCEA, they were assigned to one of 4 groups: local anesthetic alone (LA), epidural single morphine administration during surgery followed by local anesthetic alone (M), local anesthetic combined with fentanyl 10 μg/h (F10), or local anesthetic combined with fentanyl 20 μg/h (F20). The primary outcome was the number of PCEA boluses used. Secondary outcomes included the use of rescue analgesia, postoperative nausea and vomiting, and postoperative pruritus.

A total of 250 parturients were analyzed. Whereas the number of PCEA boluses in the LA group was significantly higher than in the other combined opioid groups on the day of surgery and postoperative day 1 (LA: 3 [1–6] and 7 [4–9] vs M: 2 [0–4] and 4 [0–7] vs F10: 1 [0–4] and 3 [0–6] vs F20: 1 [0–3] and 2 [0–8], *P* = .012 and 0.010, respectively), within the combined opioid groups, the number was not significantly different. Significantly fewer patients in the F20 group required rescue analgesia on postoperative day 1 and 2 (25 and 55%) than those in the M (66 and 81%) and F10 (62 and 66%) groups (*P* < .001 and *P* = .007, respectively). Postoperative nausea and vomiting and pruritus were significantly higher in the M group (*P* < .008 and *P* = .024, respectively).

The results of the present study suggest that local anesthetic alone after a single administration of morphine, or local anesthetic combined with fentanyl 10 μg/h would generally be adequate for PCEA, whereas local anesthetic combined with fentanyl 20 μg/h would be suitable for conventional epidural analgesia.

## 1 Introduction

The benefits of adequate postoperative pain relief are well established.^[1,2]^ Successful postoperative pain management is particularly important after cesarean section delivery because pain can interfere with the mother's lactation and breastfeeding and have other potential effects on the newborn.^[1,2]^ Pain relief must be rapid and effective, with minimal adverse effects on both the mother and the baby. Many methods have been used in efforts to manage postoperative pain after cesarean section, including intravenous patient-controlled analgesia, intrathecal and epidural opioids with and without local anesthetics, and patient-controlled epidural analgesia (PCEA).^[1–4]^

Epidural opioids have been widely used to facilitate central neuraxial blockade and postoperative analgesia. Multiple studies have shown that analgesia is more effective when opioids are used in combination with local anesthetics.^[5–7]^ These combinations minimize the risks of side-effects associated with solitary narcotic use such as respiratory depression, somnolence, and pruritus, while also reducing the incidences of motor block and hypotension, which are both associated with local anesthetics. More importantly, this multimodal approach has been shown to provide superior postoperative analgesia to that of intravenous patient-controlled analgesia with opioids.^[7]^ Notably however, there are few reports regarding optimal combined opioid and local anesthetic regimens in PCEA for post-cesarean section pain relief. The aim of the present study was to assess the efficacy of combined opioids by comparing 4 different PCEA pain management regimens after cesarean section.

## 2 Methods

This retrospective study was approved by the relevant hospital ethics committee for human studies (Ethics Committee Number O-0105), and the requirement to obtain informed consent was waived. A total of 404 pregnant women who had undergone elective or emergent cesarean section under combined spinal and epidural anesthesia between April 2013 and March 2016 were included in the study. The exclusion criteria were use of intravenous and/or intrathecal opioids as anesthesia adjuvants (*n* = 59), hysterectomy following cesarean section (*n* = 2), interrupted PCEA on the day of surgery (*n* = 3), and lack of medical records documenting PCEA bolus administration (*n* = 90). All patients received combined spinal-epidural anesthesia for cesarean section in a lateral position. First, after accessing the epidural space with an 18-G Tuohy epidural needle using the loss of resistance technique, an epidural catheter was inserted 3 to 5 cm from the end of the Tuohy needle into the epidural space at Th11/Th12 to L2/L3. A 25- or 27-G spinal needle was then inserted into the subarachnoid space at either the L2/L3 or L3/L4. After successful cerebrospinal fluid recognition, 1.0 to 2.0 mL of hyperbaric bupivacaine 0.5% was injected into the subarachnoid space, and the spinal needle was then removed. After a successful cold or pinprick test, the operation was initiated. In cases of inadequate levels of anesthesia, additional local anesthetic was administered via the epidural tube.

Patients received PCEA via one of the following four regimens: local anesthetic alone (0.2% ropivacaine or 0.25% levobupivacaine; LA group), epidural single morphine 1.5 to 3.0 mg administration during surgery followed by local anesthetic alone (M group), local anesthetic combined with fentanyl 10 μg/h (F10 group), and ocal anesthetic combined with fentanyl 20 μg/h (F20 group). All PCEA settings were at the basal rate, that is, 4 or 6 mL/h, with a bolus dose of 3 mL and lockout interval of 60 minutes. The PCEA device was a COOPDECH Balloonjector 300 PCA Set (Disposable Infusion Pump, Daiken Medical Co. Ltd., Izumi, Japan). When patients complained about postoperative pain, rescue analgesics including acetaminophen, flurbiprofen, and pentazocine were administered. The epidural catheter was removed postoperatively based on the decision of the attending doctor.

The primary outcome of the study was the number of PCEA boluses used on the day of the operation and on postoperative day (POD) 1. Although it would have been valuable to evaluate the adequacy of analgesia by using either a visual analogue scale or numerical rating scale to assess postoperative pain at different postoperative time points, these were not routinely recorded in our institute. Secondary outcomes included rescue analgesia, postoperative nausea and vomiting (PONV), and postoperative pruritus.

Statistical analyses were performed using JMP 11 (SAS Institute Inc., Cary, NC). Data are expressed as means ± the standard deviation, medians and interquartile ranges, or numbers. The normality of data was analyzed by Shapiro–Wilk test. For multigroup comparisons, normally distributed data were analyzed via 1-way analysis of variance. Non-normally distributed data were analyzed via the Kruskal–Wallis test, and categorical data were analyzed via Fisher exact test. *P* < .05 was deemed to indicate statistical significance. G∗power software^[8]^ (version 3.1.9.3; Dusseldorf University, Dusseldorf, Germany) was used to confirm the adequacy of the number of participants. The result of post-hoc power analysis was 0.92 based on an α value of 0.05, a total sample size of 250, and an effect size of 0.25.

## 3 Results

A total of 250 parturients were analyzed. Demographic and baseline data pertaining to the four groups are summarized in Table 1. Outcomes are shown in Table 2. The number of PCEA boluses used in the LA group was significantly higher than the other combined opioid groups on the day of surgery and POD 1; however, within combined opioid groups, the number of PCEA boluses used was not significantly different. The durations of PCEA in the opioid combination groups were significantly longer than that in the LA group, and the duration of PCEA in the F20 group was significantly longer than the durations in the other groups. This was due to lower frequency of PCEA bolus use.

**Table 1:** Patient characteristics.

	LA group	M group	F10 group	F20 group	
	*n* = 32	*n* = 149	*n* = 29	*n* = 40	*P*
Age, y	33 ± 5	33 ± 5	33 ± 4	33 ± 5	.98
Height, cm	156.4 ± 5.1	156.9 ± 5.2	158.4 ± 4.9	157.3 ± 5.1	.40
Weight, kg	61.8 ± 12.2	61.4 ± 9.1	59.7 ± 6.3	60.7 ± 7.2	.76
ASA (1/2/3/1E/2E/3E), *n*	6/7/0/5/14/0	38/47/2/31/29/2	7/6/1/6/9/0	14/14/0/5/7/0	.32
Duration of surgery, min	71 ± 17	79 ± 22	77 ± 33	78 ± 20	.47
Duration of anesthesia, min	89 ± 21	97 ± 25	94 ± 37	97 ± 23	.31
PCEA dosing rate, *n* (4/6 mL/h)	31/1	147/2	29/0	40/0	.50
Type of LA (0.2% ROP/0.25% LBUP), *n*	30/2	142/7	27/2	39/1	.82

**Table 2:** Outcomes.

	LA group	M group	F10 group	F20 group	
	*n* = 32	*n* = 149	*n* = 29	*n* = 40	*P*
PCEA bolus, median (IQR)					
Day of surgery	3.0 (1.0–6.3)	2.0 (0–4.0)^∗^	1.0 (0–4.0)^∗^	1.0 (0–3.0)^∗∗^	.012
POD 1	7.0 (3.8–9.3)	4.0 (0–7.0)^∗∗^	3.0 (0–6.0)^∗^	2.0 (0–8.0)^∗∗^	.010
POD 2	1.5 (0–4.0), *n* = 8	1.0 (0–2.0), *n* = 73	0 (0–1.0), *n* = 16	1.0 (0–2.0), *n* = 33	.190
Duration of PCEA					
(POD 1/POD 2/POD 3), *n* (%)	24^††^/8^††^/0 (75/25/0)	76/73/0 (51/49/0)	13/15/1 (45/52/3)	7^††^/32^††^/1 (18/80/2)	<.001
Patients required rescue analgesia^‡^, *n* (%)					
Day of surgery	9^††^ (28)	13 (9)	1 (3)	3 (8)	.014
POD 1	27^††^ (84)	92 (66)	18 (62)	8^††^ (25)	<.001
POD 2	23 (72)	121^††^ (81)	19 (66)	22^††^ (55)	.007
POD 3	17 (53)	77 (52)	18 (62)	24 (60)	.64
During PCEA	13 (41)	45 (32)	7 (24)	6 (15)	.080
PONV (day of surgery to POD 1), *n* (%)	1 (3)	25^††^ (18)	2 (7)	1 (3)	.008
PONV with or without prophylactic(s)^§^					
(None/1/2 types), *n* (% of each type)	1/0/0 (6/0/0)	20/6/0 (16/17/0)	1/2/0 (20/7/0)	1/1/0 (11/4/0)	—
None/1/2 medication(s), *n*	18/14/0	111/35/3	5/23/1	9/26/5	—
Pruritus (day of surgery to POD 1), *n* (%)	0	21^†^ (14)	2 (7)	4 (10)	.024
Pruritus with or without prophylactic(s)^||^					
(None/one/two types), *n* (% of each type)	0/0/0	20^¶^/0/0 (16/0/0)	1/1/0 (8/7/0)	1/3/0 (10/12/0)	—
None/1/2 medication(s), *n*	28/4/0	25/23/11	13/15/1	10/25/5	—

The number of patients who required rescue analgesia in the LA group was significantly larger than that in the opioid combination groups on the day of surgery and POD 1. Within the combined opioid groups, significantly fewer patients required rescue analgesia in the F20 group on POD1 and 2 than in the M and F10 groups. The number of patients who required rescue analgesia while using PCEA tended to decrease in the opioid combination groups (LA > M > F10 > F20).

The incidence of PONV was significantly greater in the M group compared to the other groups. The use of prophylactic medication was inversely associated with PONV in the F10 and F20 groups, but not in the M group. The incidence of postoperative pruritus was greater in the opioid combination groups; within the combined opioid groups, the incidence of pruritus was significantly greater in the M group when compared to that of the other groups. Pruritus was also more severe in the M group than it was in the F10 and F20 groups.

## 4 Discussion

The combination of local anesthetics and opioids has the benefit of achieving postoperative analgesia without substantial motor blockade, which is extremely important for mothers with respect to their capacity to take care of their babies after cesarean section.^[2]^ Epidural morphine is a well-established standard medication for post-cesarean section pain relief.^[9,10]^ A single dose of epidural morphine provides more effective analgesia than systemic opioid administration.^[9]^ It has also been reported that fentanyl has a sparing effect on the dose of local anesthetic when the 2 are combined.^[11,12]^

In the present study, the combination of opioids with local anesthetics was more effective for analgesia after cesarean section than local anesthetic alone, as indicated by the lower use of PCEA bolus on the day of surgery and on POD 1 in the opioid combination groups compared to the LA group. Within the opioid combination groups, bolus frequency tended to be lower in the F20 group than in the M and F10 groups on the day of surgery and POD 1, which suggests that PCEA lasted the longest in the F20 group. Although bolus times were lower on POD 2, PCEA was discontinued on POD 2 in some patients in every group, most frequently in the M group, whereas rescue analgesic requirements were either maintained or increased, indicating that severe postoperative pain continued up to POD 2. Thus, the addition of opioids to local anesthetic or an increase in the basal dosing rate of PCEA may be required for early-phase postoperative analgesia.

Rescue analgesic requirements were lowest in the F20 group, sequentially followed by the F10, M, and LA groups (F20 < F10 < M < LA). This might reflect opioid dose, because epidural local anesthetic administration is effective for postoperative wound pain, but less effective for postpartum pain associated with uterine contractions. When local anesthetic alone is chosen in epidural analgesia, scheduled administration of nonsteroidal anti-inflammatory drugs or acetaminophen will be helpful for postoperative analgesia. Although there were no significant differences in the numbers of PCEA boluses used within the opioid combination groups, the bolus frequency and number of patients who used rescue analgesia during PCEA were lowest in the F20 group. These findings suggest that local anesthetic combined with fentanyl 20 μg/h may be a better choice for conventional epidural analgesia.

PONV was significantly higher in the M group than in the other groups. The combined use of 2 prophylactics such as hydroxyzine hydrochloride and haloperidol would be effective for the prevention from PONV due to opioids.^[13]^ Pruritus was also more severe in the M group than it was in the F10 and F20 groups, indicating that the prophylactic medication failed to reduce pruritus in the opioid combination groups. There are 2 types of pruritus derived from opioids, central and peripheral. Central pruritus involves the central nervous system μ opioid receptor, whereas peripheral pruritus is caused by histamine release from stimulated mast cells.^[14]^ Because epidural opioid administration causes central pruritus via the spinal μ receptor—especially morphine, which is highly water soluble—antihistamine or droperidol are not effective for prophylaxis.

The present study has several limitations. Pain assessment via methods such as a visual analog scale was not performed routinely in our institute. PCEA push times during lockout intervals were not considered. Lastly, the total consumption of local anesthetics and opioids was not investigated.

In conclusion, the combination of local anesthetics and opioids is advantageous for postoperative analgesia after cesarean section, although PONV and pruritus should be prevented. Our results suggest that local anesthetic alone after a single administration of morphine, or local anesthetic combined with fentanyl 10 μg/h would generally be adequate for PCEA, whereas local anesthetic combined with fentanyl 20 μg/h would be suitable for conventional epidural analgesia. However, further prospective studies with large sample sizes are needed to validate the results of the present study and to identify optimal PCEA settings such as background infusion rate of local anesthetics and opioid use.

## Acknowledgments

This study was attributed to Departments of Anesthesiology, University of Miyazaki Hospital. The authors thank Mio Kurogi, Noriko Hidaka, and Toshiko Watanabe (research assistant and secretary belong to Department of Anesthesiology) for their assistance with data collection. We also thank Editage (www.editage.jp) for English language editing.

## Author contributions

GO and TM designed and conducted the study, analyzed the data, and wrote the manuscript. IT conceived the study and participated in study design and coordination. All authors have read and approved the final manuscript.
